# Climate Change Projected Effects on *Hamatocaulis vernicosus* Occurrence in Romania

**DOI:** 10.3390/plants14213354

**Published:** 2025-10-31

**Authors:** Sorin Ștefănuț, Claudia Biță-Nicolae, Tiberiu Sahlean, Constantin-Ciprian Bîrsan, Ioana Cătălina Paica, Georgiana-Roxana Nicoară, Florența-Elena Helepciuc, Miruna-Maria Ștefănuț, Ana-Maria Moroșanu

**Affiliations:** Institute of Biology Bucharest, Romanian Academy, 296 Splaiul Independenței, P.O. Box 56-53, 060031 Bucharest, Romania

**Keywords:** *Hamatocaulis vernicosus* (Mitt.) Hedenäs, bryophytes, mosses, EU Habitats Directive, climate change, threatened species

## Abstract

*Hamatocaulis vernicosus* is a pleurocarpous moss of conservation concern, listed in Annex II of the EU Habitats Directive due to its significant and ongoing decline across Europe. *H. vernicosus* is also listed as ‘Vulnerable’ on the Red List of Romanian Bryophytes. Despite its protected status, the species remains under-recorded in Romania, where many potentially suitable habitats have yet to be surveyed. The ecosystems, classified as Transition mire and quaking bog (NATURA 2000 code: 7140), are wet peatlands with oligo- to mesotrophic conditions and a pH of 5.0–7.5 H. vernicosus is recorded in 58 Romanian locations (10 confirmed by us, 5 new), spanning the Continental and Alpine bioregions. Models showed good performance (AUC 0.79–0.83; TSS 0.54–0.59), with distribution mainly shaped by mean annual temperature and temperature range, and secondarily by precipitation. The species favors cold, stable climates with high seasonal rainfall. Even though the number of localities reported for this species has increased in recent years, this does not indicate an improvement in its conservation status, but rather is an effect of recent recording efforts. To support targeted conservation planning, an ensemble species distribution model was developed in order to predict the suitable habitats of *H. vernicosus* across Romania. Both climate models project major range losses for the varnished hook-moss: ~30% by 2050 and ~40–60% by 2100, depending on the scenario. Losses are gradual under SSP245 but more abrupt under SSP585, with increased fragmentation, especially between the Eastern and Southern Carpathians. By integrating field observations with predictive climate change modeling, our study brings critical insights applicable to the conservation of *H. vernicosus* and the unique peatland ecosystems it relies on.

## 1. Introduction

*Hamatocaulis vernicosus* (Mitt.) Hedenäs is a medium-sized pleurocarpous moss, commonly found in mesotrophic meadows. It typically forms green to yellowish-green carpets with distinctive hooked shoot tips and strongly curved, plicate leaves that are often reddish at the base [1]. The species is dioicous and reproduces asexually by fragmentation [2]. Formerly known as *Drepanocladus vernicosus*, it was recombined in the genus *Hamatocaulis* by Hedenäs in 1989 [3]. Although it can be confused with similar mosses, it can be reliably distinguished by key anatomical features. Genetic studies have identified two cryptic clades of *H. vernicosus* with overlapping distributions in Europe and North America, although no corresponding morphological differences have been observed [4,5].

**Figure 1:**
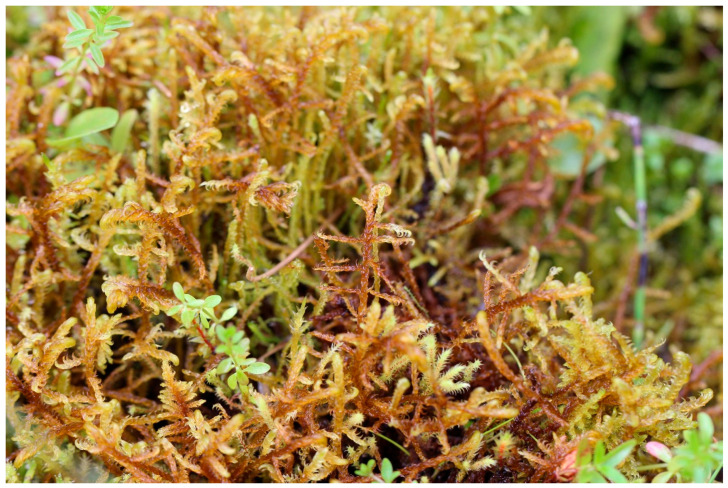
*Hamatocaulis vernicosus* from Horoaba Valley, Bucegi Mountains, 10.08.2016.

*H. vernicosus* (Figure 1) is a circumboreal species occurring in temperate and arctic northern temperate zones, with a restricted distribution in Europe, Asia, and North America [6,7]. *H. vernicosus* is distributed across several regions in Europe and beyond, though its presence is often threatened or declining [8]. It is recorded in parts of the Caucasus and Central Asia, including Georgia, Armenia, Kazakhstan, Kyrgyzstan, and Russia [9]. In Eastern Europe, it occurs in Belarus and Ukraine, and it is also found in the Balkans, including Bosnia-Herzegovina, Macedonia, Montenegro, Slovenia, and Serbia [10,11]. The species is still present locally in areas of Germany and Switzerland, while in the UK, its distribution is scattered and declining [12,13,14]. It has declined significantly in countries such as the Czech Republic, France, and Spain, mainly due to wetland degradation and land-use changes [15,16,17]. *H. vernicosus* is also found in the Faroe Islands [18]. It has become extinct in Luxembourg and has disappeared from Hungarian wet meadows due to eutrophication and drainage [19].

*H. vernicosus* typically inhabits intermediate fens and spring-fed flushes influenced by mineral-rich, yet not strongly calcareous, groundwater [20]. It is most often found in upland base-rich springs or lowland small-sedge fens with mildly basic conditions. These habitats, classified under the EU Habitats Directive as *Transition mires and quaking bogs* (code 7140), are wet peatlands characterized by oligo- to mesotrophic nutrient levels and a pH range of approximately 5.0 to 7.5 [21]. *H. vernicosus* is protected under multiple European conservation frameworks, including Appendix I of the Bern Convention [22] and Annex IIb of the EU Habitats Directive [23]. It is listed as ‘Vulnerable’ in the Red Data Book of European Bryophytes and is considered a qualifying interest in Special Areas of Conservation (SACs) across Europe [24]. *H. vernicosus* is also listed as ‘Vulnerable’ in Europe [25] and on the Red List of Romanian Bryophytes [26].

The aims of this study are to assess the current and potential distribution of *Hamatocaulis vernicosus* in Romania, to evaluate the projected effects of climate change on its occurrence using ensemble species distribution modeling, and to provide insights into species-specific responses to environmental change. By linking field observations with predictive modeling, this study seeks to support targeted conservation planning and effective management of transition mires and quaking bogs, thereby contributing to the long-term protection of vulnerable peatland.

## 2. Results

### 2.1. Current Species Distribution

**Figure 2:**
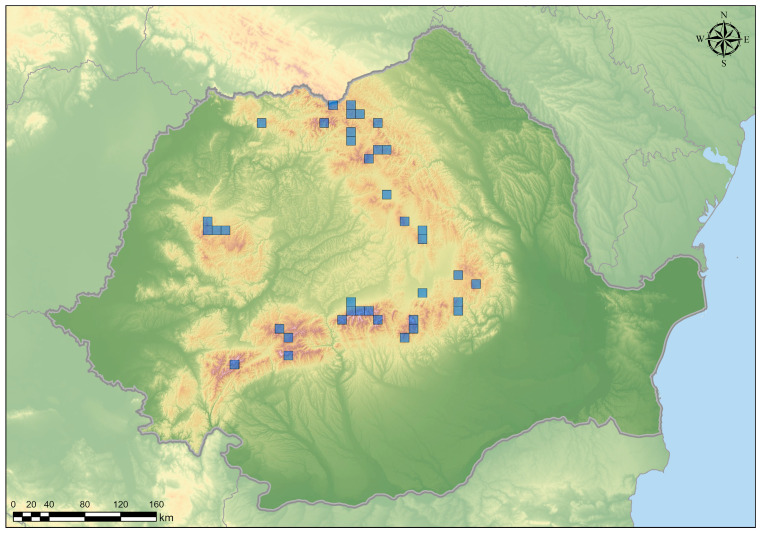
Distribution map for the Slender Green Feather Moss (*Hamatocaulis vernicosus*). Grid cells used for distribution mapping are reference ETRS89–LAEA grids available from the European Environmental Agency.

The current distribution of *H. vernicosus* in Romania (Figure 2, Supplumentary Material):

Maramureș County, Mlaștina de la Budești [27];

Maramureș Mountains, Fântâna Stanchi, 47.664750° N, 24.877470° E, 1687 m a.s.l., 09 August 2014, leg. Hájková P. & Hájek M., 2014/037, det. Hájková P. and Hájek M. [BRNU 680073], leg. Hájková P. and Hájek M., 2014/039, det. Hájková P. and Hájek M. [BRNU 680075] [28];

Rodna Mountains, *without location* [29];

Bistrița-Năsăud County, Gradinița Peatbog [30,31];

Suceava County, Teșna Românești Peatbog [32,33,34]; Poiana Stampei Peatbog [35]; Lucina-Găina Peatbog [30], 1161 m a.s.l., 3–10 May 1972 [36]; Camionca–Lucina Peatbogs, 1226 m a.s.l., 1–4 September 1953, leg. and det. Ștefureac T. [33]; Drăgoiasa Peatbog, 1064 m a.s.l., 1959, leg. and det. Ștefureac T. [37]; Valea Stânii Peatbog, 1107 m a.s.l. [38]; Cristișor Peatbog, 808 m a.s.l. [39]; Botuș, 47.573890° N, 25.349530° E, 802 m a.s.l., 08 August 2014, leg. Hájková P. and Hájek M., 2014/035, det. Hájková P. and Hájek M. [BRNU 680071] [28];

Rarău Mountain, Chirilu brook [40]; Codrul Secular Slătioara, 1350–1400 m a.s.l. [40,41]; Plaiul Todirescu, 1367 m a.s.l. [42,43];

Călimani Mountains, the peatbog from Răchitișul Mare de Sus, 23–24.07.1972, *leg*. Coldea G., *det*. Plămadă E. [44];

Țibleș Mountains, Mesteacănului Valley, Țibleș Valley [45];

Harghita County, Depresiunea Giurgeului, Bazinul Ciuc, Pietroasa Peatbog (Joseni), Bazinul Gheorghieni [30]; Szökő Peatbog, 1609 m a.s.l. [46,47,48]; Tușnadu Nou, 46.199500° N, 25.890028° E, 640 m a.s.l., 01.08.2013, *det*. B. Papp [BP *187892*] [28]; Tușnadu Nou, Varsavesz, wetland between the railway and Olt River, 46.199667° N, 25.890806° E, 638 m a.s.l., 01.08.2013 *leg*. and *det*. Papp B.; Sânsimion, 46.256110° N, 25.856640° E, 646 m a.s.l., 05 August 2014, *leg*. Hájková P. and Hájek M., 2014/007, *det*. Hájková P. and Hájek M. [BRNU *680043*] [28]; Sânsimion, Felso Honcsok wetland, 46.257361° N, 25.854694° E, 647 m a.s.l., 31 July 2013, *leg*. and *det*. Papp B. [BP *187849*] [49]; Vrabia, 46.216140° N, 25.890140° E, 641 m a.s.l., 05.08.2014, *leg*. Hájková P. and Hájek M., 2014/011, *det*. Hájková P. and Hájek M. [BRNU *680047*] [28];

Covasna County, Comandău Peatbog [50];

Penteleu Mountains, Penteleu [51];

Siriu Mountains, western slope of Mălâia [52]; Lacul Sec Peatbog, 7 Octomber 2021, *det*. Ștefănuț S.

**Figure 3:**
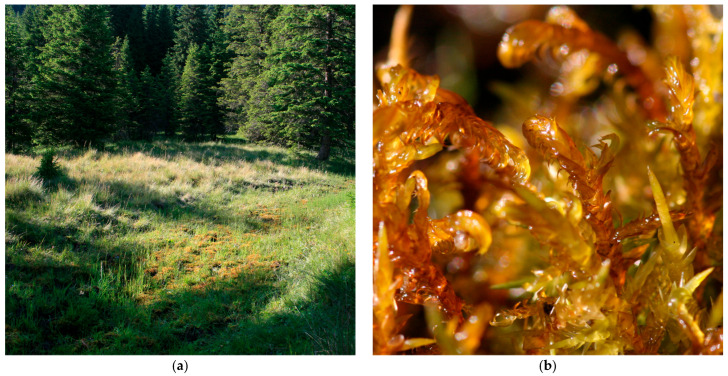
*Hamatocaulis vernicosus* in Bucegi Massif, Romania, 10 August 2016: (**a**) Habitat of *H. vernicosus,* Horoaba Valley, 1521 m a.s.l.; (**b**) *Hamatocaulis vernicosus*–close up.

**Figure 4:**
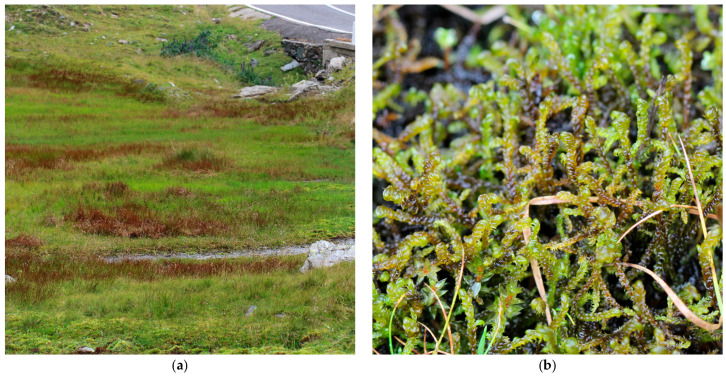
*Hamatocaulis vernicosus* in Făgăraș Mountains, Romania, 4 August 2016: (**a**) Habitat of *H. vernicosus,* Bâlea Valley, 1925 m a.s.l.; (**b**) *Hamatocaulis vernicosus*–close up.

Brașov County, Hărman Peatbog, 45.717676° N, 25.669150° E, 518 m a.s.l. [53];

Prahova County, Bâlbâitoarea Peatbog [54];

Bucegi Mountains, Dorului valley, Blana, Nucet and Lăptici Mountains, 1800 m a.s.l., det. Ștefureac T. [55]; Horoaba Valley, Dâmbovița County, N 45.390722°, E 25.433694°, 1521 m a.s.l.; 6.09.2022; *det*. Ștefănuț S.; Lăptici Peatbog, Dâmbovița County, N 45.371806°, E 25.436306°, 1468 m a.s.l.; 6.09.2022; det. Ștefănuț S. and Tamas G. [56]; Horoaba Valley, 2008, 11 July 2011, 10 August 2016, *det*. Ștefănuț S., Nicoară R. (Figure 3); Nucet Mountain, Dorului Valley, Bucegi Mountains, 45.356608° N, 25.462988° E, 1779 m a.s.l., 12 June 2025, *det*. Ștefănuț S., Bîrsan C.-C., Ștefănuț M.-M. and Moroșanu A.-M.; Lăptici Peatbog, 12 June 2025, *det*. Ștefănuț S., Moroșanu A.-M., Bîrsan C.-C. and Ștefănuț M.-M.; Scândurilor Valley Peatbog, Dâmbovița County, 45.373407° N, 25.437636° E, 1476 m a.s.l., 10 May 2010, 10.08.2016, 12 June 2018, *det*. Ștefănuț S., 45.373653° N 25.437814° E, 1476 m a.s.l., 12 June 2025; *det*. Ștefănuț S., Moroșanu A.-M., Bîrsan C.-C. and Ștefănuț M.-M.;

Leaota Mountains, Vaca Valley [57];

Făgăraş Mountains, Doamnei Valley, *leg*. Nyárády E.I., *det*. Papp C. [58]; Bâlea glacial ring, *leg*. Diaconeasa B., *det*. Ștefureac T., as *Drepanocladus* [59]; Bâlea glacial ring, 45.607034° N, 24.617185° E, 1925 m a.s.l., 04.08.2016, 7.11.2024, *det*. S. Ștefănuț; Sărata glacial ring, *leg*. Diaconeasa B., *det*. Ștefureac T., sub *Drepanocladus* [60]; Capra glacial ring, *leg*. Diaconeasa B., *det*. Ștefureac T., sub *Drepanocladus* [61]; Capra glacial ring, the peatbog near to Capra Lake 45.599414° N 24.628372° E, 2234 m a.s.l., 25 August 2017, *det*. S. Ștefănuț (Figure 4); Puha I, 2015 m a.s.l., Puha II, 1882 m a.s.l., *leg*. Diaconeasa B., *det*. Ștefureac T., sub *Drepanocladus* [62]; Ucea-Corabia glacial ring, 1856 m a.s.l., *leg*. Diaconeasa B., *det*. Ștefureac T., sub *Drepanocladus* [63]; near Zârna Lake, 45.589408° N, 24.861041° E, 2058 m a.s.l., 24 July 2019, *det*. S. Ștefănuț;

Iezer-Păpușa Mountains, Iezer [56], the peatbogs from Iezer Lake, *det.* Ștefănuț S., 8.08.2024;

Sibiu County, Mlaca Tătarilor Peatbog, 45.715197° N, 24.649820° E, 540 m a.s.l., 29 September 2016 *det*. Ștefănuț S.; 45.715140° N, 24.650140° E, 540 m a.s.l., 05.08.2018, *leg*. Hájková P. and Hájek M., 2018/141, *det*. Hájková P. [BRNU 680374] [28];

Cindrel Mountains, Frumoasa, 1330 m a.s.l., 30 July 2018, *leg*. Hájková P. and Hájek M., 2018/068, *det*. Hájková P. [BRNU *680302*], 1328 m a.s.l., 30 July 2018, *leg*. Hájková P. and Hájek M., 2018/066, *det*. Hájková, P. [BRNU *680300*] [28];

Lotru Mountains, near Larga Mânețanilor, 45.481690° N 23.691920° E, 1702 m a.s.l., 3 August 2018, *leg*. Hájková P. and Hájek M., 2018/122, *det*. Hájková P. [BRNU *680356*], 45.482000° N, 23.693140° E, 1708 m a.s.l., 3 August 2018, *leg*. Hájková P. and Hájek M., 2018/124, *det*. Hájková P. [BRNU *680358*] [28]; Tampa, 45.456190° N, 23.647530° E, 1783 m a.s.l., 3 August 2018, *leg*. Hájková P. and Hájek M., 2018/109, *det*. Hájková P. [BRNU *680343*], 45.455920° N, 23.647530° E, 1781 m a.s.l., 3 August 2018, *leg*. Hájková P. and Hájek M., 2018/112, *det*. Hájková P. [BRNU *680346*] [28]; Bahnele de la Tărtărău, 45.508110° N, 23.650360° E, 1340 m a.s.l., 2 August 2018, *leg*. Hájková P. and Hájek M., 2018/097, *det*. Hájková P. [BRNU *680331*] [28];

Șureanu Mountains, Luncile Prigoanei, 45.586110° N 23.571670° E, 1390 m a.s.l., 2 August 2018, *leg*. Hájková P. and Hájek M., 2018/101, *det*. Hájková P. [BRNU *680335*] [28];

Parâng Mountains, Câlcescu Peatbog [64];

Retezat Mountains, towards Tăul Judele [65];

Cluj County, Dâmbu Negru - La Pod Peatbog [66]; Dâmbu Negru–Călățele, 3.06.1936, 26 July 1937, *leg*. Pop E., *det*. Ștefureac T. [67]; Beliș-Dâmbu Negru, 46.695353° N 23.037403° E, 1087 m a.s.l, 15 Octomber 2006, *det*. Pócs T., Buczkó K., 06083/B [BP *177092*] [28]; Mlaștina deasupra Lacului Fântânele, in poor fen, 46.668952° N, 22.965863° E, ca 1178 m a.s.l. [68];

Vlădeasa Mountains, Vârfuraș, Micău, Rogojel, *leg*. Resmeriță I., *det*. Ștefureac T. and Plămadă E. [69];

Bihor County, Padiș, in *Sphagnum* peat bog, 2–9 July 1961, *leg*. and *det*. Páll Ș. [70];

Bihor Mountains, *without location* [71].

### 2.2. Species Distribution Model Performance

**Figure 5:**
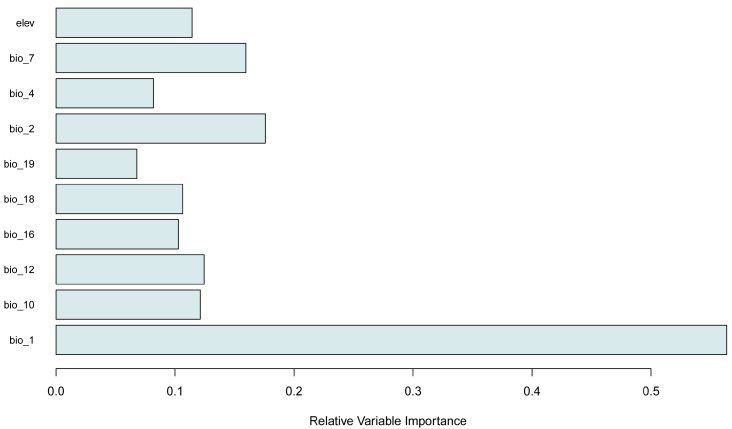
Relative variable importance for all models across all modeling methods.

All models performed well, with mean AUC values between 0.79 and 0.83, and mean TSS values from 0.49 to 0.59 (Table 1). The highest contributing variables were Annual Mean Temperature (bio_1, 56.4%), Mean Diurnal Range (bio_2, 17.6%), and Temperature Annual Range (bio_7, 16%) (Table 2, Figure 5).

**Figure 6:**
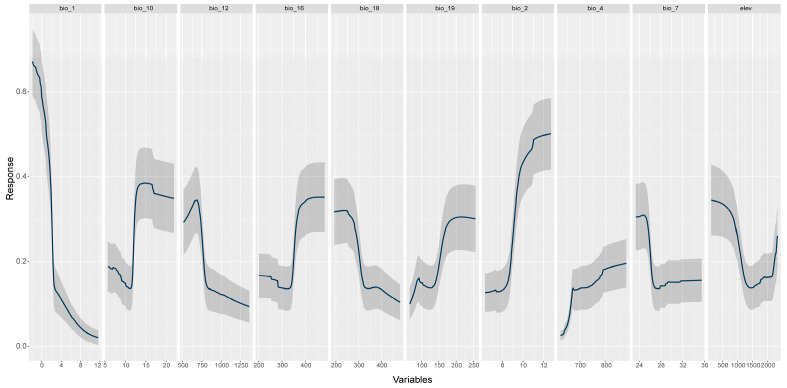
The response curves for all predictors used to generate the SDMs.

The response curves (Figure 6) coupled with the variable’s contributions (Figure 5), suggest that the species’ range is limited primarily through the influence of the mean annual temperature and the Annual Temperature Range. The distribution range of the varnished hook-moss consists of areas characterized by low mean annual temperature, generally below 4 °C (Figure 5), high amplitudes of diurnal temperature variation (up to 12 °C), and low variation of annual temperature, below 26 °C (Table 2, Figure 5 and Figure 6).

### 2.3. Current Potential Distribution

**Figure 7:**
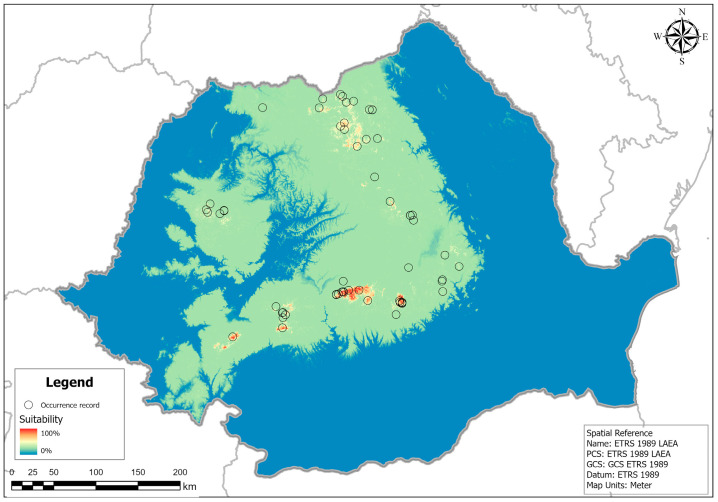
The ensemble habitat suitability model for the varnished hook-moss (*Hamatocaulis vernicosus*). Blue shades indicate low suitability, while red shades signify high suitability.

**Figure 8:**
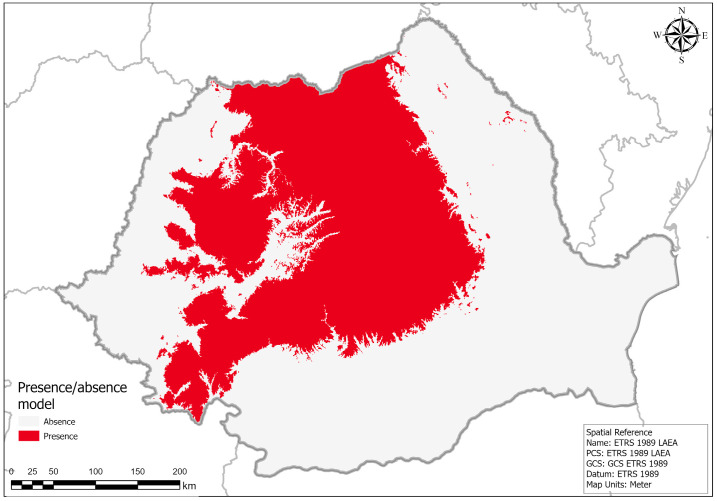
Presence–absence map generated using the threshold value maximizing TSS across the test data.

According to the ensemble model and the threshold models (Figure 7 and Figure 8), the current potential distribution for *H. vernicosus* in Romania is limited to the Carpathian Mountains, parts of the hilly region below the Carpathians, and a large part of Transylvania. However, areas with high suitability are limited to a few mountainous areas in the Eastern and Southern Carpathians, while in the Western-Romanian Carpathians there are only some areas of moderate suitability in the western part of the Apuseni Mountains.

### 2.4. Future Distribution

**Figure 9:**
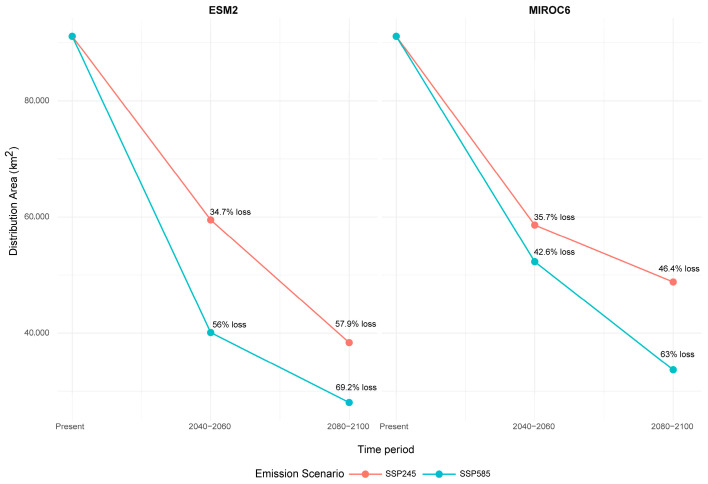
Predicted changes in the distribution range of the varnished hook-moss (*Hamatocaulis vernicosus*).

Both global circulation models agreed on a drastic loss of distribution space for the varnished hook-moss by the end of the century. The predictions differ somewhat between the CMCC–ESM2 and the MIROC6 models (smaller differences for the mid-century projections and greater differences for the end-century projections), but they agree on an average loss of about 30% in habitable space by the end of 2050 in the most likely socioeconomic scenario, and a ~40–50% loss in the most pessimistic case (SSP585). By the end of the century, even in the most likely socioeconomic scenario, the species is expected to lose around ~40–50% of its habitable range; that increases to around 60% in the worst-case scenario (Table 3, Figure 9).

**Table 3:** Predicted suitable area (in km^2^) for present and future climatic conditions and emission scenarios and predicted loss of environmental space for the species.

		CMCC–ESM2			
Period	Present	2040–2060		2080–2100	
Emission scenario	-	SSP245	SSP585	SSP245	SSP585
Area (in km^2^)	91,125.98	59,500.04	40,099.61	38,368.91	28,054.93
Loss (in km^2^)		31,625.94	51,026.37	52,757.07	63,071.05
		MIROC6			
**Period**		**2040–2060**		**2080–2100**	
Emission scenario		SSP245	SSP585	SSP245	SSP585
Area (in km^2^)		58,612.27	52,296.24	48,813.41	33,674.49
Loss (in km^2^)		32,513.71	38,829.74	42,312.57	57,451.49

**Figure 10:**
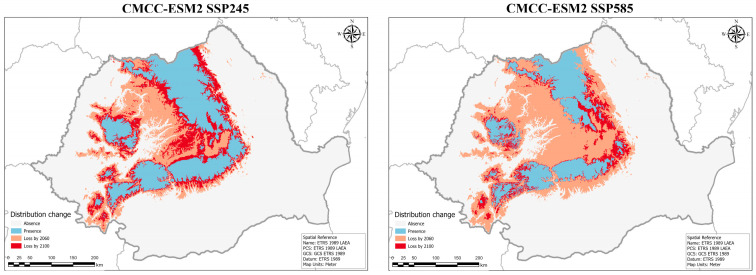
Predicted distribution range changes for the varnished hook-moss (*Hamatocaulis vernicosus*) by 2100 based on the CMCC–ESM2 SSP245 global circulation models.

**Figure 11:**
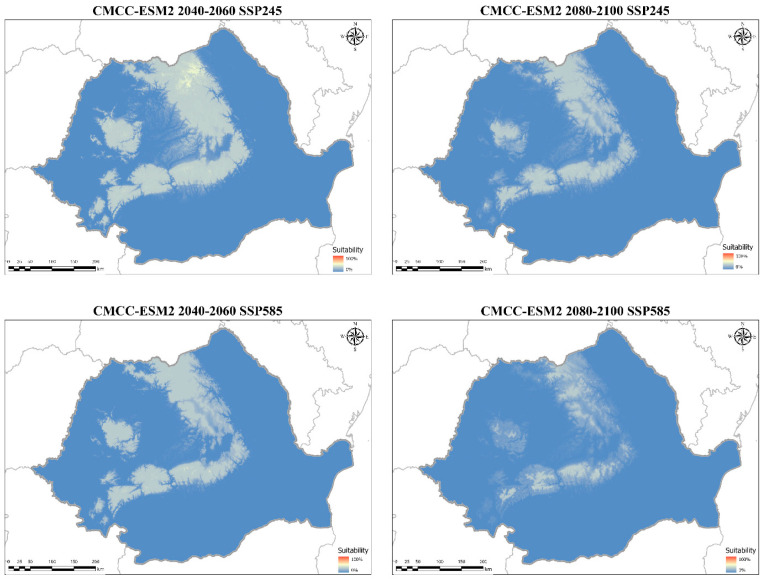
Ensemble future habitat suitability models for the varnished hook-moss (*Hamatocaulis vernicosus*) based on the CMCC–ESM2 global circulation models for two time periods and two socioeconomic pathways. Blue shades indicate low suitability, while red shades signify high suitability.

In the CMCC–ESM2 SSP245 scenario the losses are more gradual by the middle of the century and towards 2100, while in the SSP585 scenario most of the loss takes place by 2060, and reduced parts of the range continue to decline until 2100 (Figure 10). Notably, from 2060 to the end of the century, the Southern Carpathians are influenced by the least amount of favorable space reduction in the SSP585 scenario. Also, in this scenario a large gap in the range develops between the Eastern and Southern Carpathians, resulting in more fragmented distribution, with swathes of uninhabitable space emerging in the Southern and Western Carpathians (Figure 11).

**Figure 12:**
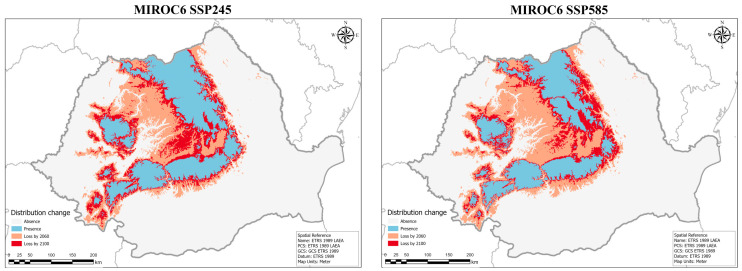
Predicted distribution range changes for the varnished hook-moss (*Hamatocaulis vernicosus)* by 2100 based on the MIROC6 SSP245 global circulation models.

**Figure 13:**
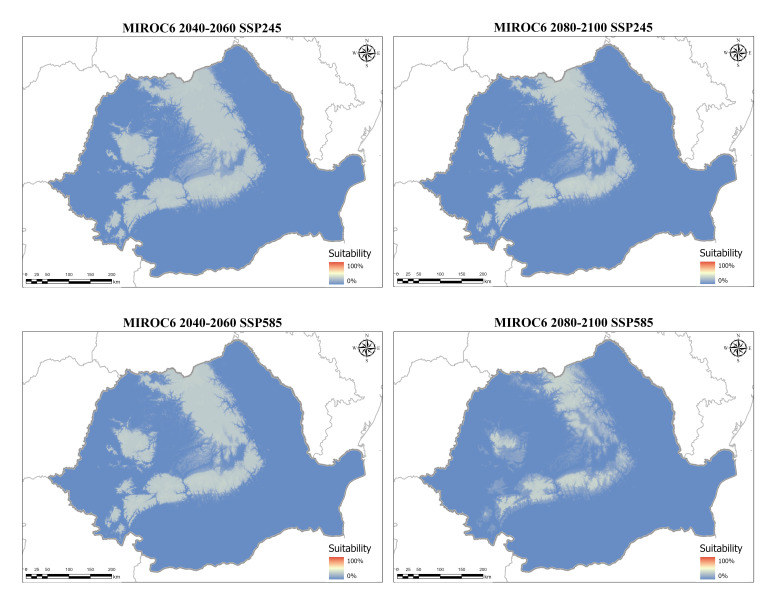
Ensemble future habitat suitability models for the varnished hook-moss (*Hamatocaulis vernicosus*) based on the MIROC6 SSP245 global circulation models for two time periods and two socioeconomic pathways. Blue shades indicate low suitability, while red shades signify high suitability.

The MIROC6 SSP245 model also predicts a gradual decrease in environmental space, with much of Transylvania losing favorable conditions through the middle of the century, followed by losses in low mountainous areas (Figure 12). Like the other community model, the SSP585 socioeconomic pathway for MIROC6 foresees an increased loss of habitable space by 2060, followed by a smaller reduction until the end of the century (Figure 13). Notably, the gaps in the range that develop are not as large as predicted by the CMCC–ESM2 model, and overall, the distribution is not as fragmented.

### 2.5. Uncertainty in Future Projections

Predicted suitable areas showed moderate variation between global circulation models. Across time periods and emission scenarios, suitable area differences ranged from 2 to 10% (Annex 1 Table S1). Spatial overlap between the two circulation models was consistently high (Schoener’s D = 0.93–0.98), indicating strong agreement in projected future distributions (Annex 1 Table S1).

## 3. Discussion

### 3.1. Distribution Patterns

The distribution of *Hamatocaulis vernicosus* in Romania was recorded in 58 locations, of which 10 were confirmed by us, and 5 locations are presented here as new records. The 58 locations of *H. vernicosus* are distributed in two bioregions of Romania, Continental and Alpine.

The habitat of *H. vernicosus* is very fragmented and very dependent on water springs. Therefore, in order to have a better vision of the evolution of the varnished hook-moss (*H. vernicosus*) distribution over time, the potential current and future species distribution models (SDMs) were developed using a combination of original and published records, comprising the occurrences in the Romanian Carpathians.

As mentioned above, the species presents regional variations in Europe, with a decline in population in western and central areas due to the disappearance of wetlands, while remaining more widespread in certain parts of Northern Europe [15,16,17,18,19,20,72,73]. Across Romania, Sweden [20], and Lithuania [73], *H. vernicosus* consistently occupies wetlands, peatlands, and fens with relatively stable hydrology. The species typically becomes settled in later successional stages, following the modification of substrates by early colonizers, and is highly sensitive to land-use changes, drainage, and other hydrological alterations. Populations tend to decline in areas that are drained, heavily grazed, or fertilized.

The number of *H. vernicosus* observations provides useful, though cautious, insights into its distribution. In both Romania and Sweden [20], areas with more records likely reflect habitats where the species is more consistently present. However, observation density is influenced by accessibility and research effort, rather than true abundance. In Sweden [20], for example, the apparent increase in recent records partly results from intensified monitoring and conservation interest rather than a genuine population growth.

*H. vernicosus* in both Romania and Lithuania [74] occurs in wetlands and fens, where its presence is closely linked the physical-chemical parameters of local water bodies, such as pH, nutrient levels, and conductivity, as well as vegetation structure. Populations are highly sensitive to local disturbances, including drainage, agriculture, and overgrazing, and are typically found in small, fragmented habitats. In contrast, in Sweden [20] the species occupies larger, more stable wetlands, and its distribution is more continuous and less tightly dependent on fine-scale water parameters, resulting in more stable long-term populations.

The species’ predicted distribution is highly fragmented, reflecting its dependence on specific hydrological and climatic conditions. Variable importance and contribution showed that the distribution is primarily driven by temperature-related factors, especially Annual Mean Temperature (56.4%), Mean Diurnal Range (17.6%), and Temperature Annual Range (16%). Other precipitation-related variables and elevation had moderate contributions, underscoring the species’ sensitivity primarily to thermal regimes.

*H. vernicosus* exhibits fragmented, human-sensitive populations across Europe, with local distribution strongly influenced by wetland hydrology, water chemistry, and land-use practices. However, these habitats are very sensitive to changes in hydrological regimes or to anthropogenic disturbances [49]. These findings are in line with earlier studies that demonstrated the species prefers stable, cool-temperature microclimates, particularly in upland areas, where these climatic conditions are present [4]. Furthermore, other authors reported vegetation changes strongly influence the diurnal temperature range, and recent vegetation increases explained its observed decline [75], as the vegetation dynamics are particularly sensitive to nighttime thermal conditions.

The ensemble potential distribution model for *H. vernicosus* in Romania indicates that the most suitable habitats are concentrated in the mountainous regions, particularly in the Southern and Eastern Carpathians, with smaller patches in the Apuseni Mountains. Areas with suitability values ≥ 70% align closely with known occurrence records but also reveal additional potentially suitable sites without confirmed records, suggesting priorities for targeted field survey. These results are consistent with ecological observations from Romania and other parts of Europe, where the species is typically confined to wetlands in stable, late-successional stages and is sensitive to hydrological alterations and land-use change. When combined with genetic data showing two cryptic lineages with overlapping ecological niches but distinct geographic distributions, the model outcomes underscore the importance of both local habitat quality and historical biogeography in shaping the species’ range. Integrating ecological, genetic, and spatial modeling approaches thus provides a robust framework for guiding conservation planning and anticipating potential range shifts under future climate scenarios.

The distribution model for *H. vernicosus* also revealed a heterogeneous distribution of suitable areas across Romania. Areas with high suitability are mainly concentrated in the Eastern and Southern Carpathians, as well as in isolated sectors of the western and southwestern regions. Areas with moderate suitability indicate potentially favorable habitats, where the presence of the species depends on specific local conditions, such as constant humidity or microhabitat structure. Regions with low suitability and unsuitable areas cover most of the Romanian Plain, Dobrogea, and other lowland areas, where environmental conditions are unfavorable for the species. Most records of presence overlap with areas of high or moderate suitability, confirming the robustness of the model, while certain areas of high suitability without records could represent priorities for future field studies and potential extensions of protected sites. The presence–absence map, generated using the threshold value that maximized the True Skill Statistic on the test data, indicates that *H. vernicosus* has a potential niche that covers areas of the Carpathian region and adjacent hills (red). These predicted presence zones encompass most of the Eastern, Southern, and Western Carpathians, as well as the Subcarpathian hills, reflecting the species’ preference for cooler, moisture-rich habitats. Areas predicted as absence zones (blue) are mainly located in lowland regions such as the Romanian Plain, Dobrogea, and other arid or intensively cultivated landscapes, where environmental conditions are unsuitable. This binary prediction highlights priority regions for conservation efforts and provides a clear framework for targeted field verification.

### 3.2. Conservation Measures

Our results (based on species distribution models–SDMs) confirm the geographic limitation of *H. vernicosus* predominantly in habitats 7140 (Transition mires and quaking bogs). Furthermore, given the vulnerable status of *H. vernicosus* and the fact that bryophytes frequently serve as bioindicators of wetland health [76], the significance of preserving these habitats extends beyond biodiversity to include ecosystem services, such as carbon sequestration and water regulation.

In this context, conservation measures, including inclusion in EU and international directives, have helped raise awareness and support protection. Its distribution is generally patchy and scattered, and long-term records indicate fluctuations in frequency that are largely linked to human activities rather than climatic factors. Similarly, Ștefănuț et al. (2023) [77] explained that they support the designation of new NATURA 2000 sites or the expansion of existing ones for *Buxbaumia viridis* in Romania, thus contributing to the European Commission’s objective in the EU Biodiversity Strategy for 2030—Bringing nature back into our lives—to protect at least 30% of Europe’s land area by 2030 [77].

Recent findings from previously unprotected sites underscore the need to expand existing NATURA 2000 boundaries and designate new sites. Conservation measures should focus on maintaining hydrological integrity, preventing eutrophication, and preserving open fen vegetation, which are essential for the long-term survival of *H. vernicosus* populations. Although both *B. viridis* and *H. vernicosus* indicate a high conservation value, *B. viridis* is more dependent on forest structure and dead wood, while *H. vernicosus* is strongly conditioned by hydrology and nutrient levels. Integrating field studies with species distribution modeling is essential for both species to identify unprotected populations, refine habitat suitability maps, and guide effective conservation planning.

### 3.3. Future Studies

The expansion of assessments in potentially suitable areas identified by SDMs for validating model predictions, alongside the integration of molecular genetic analyses and continued long-term monitoring of *H. vernicosus* population trends linked to climate changes, are all critical future research priorities. These combined approaches will strengthen the development of improved conservation strategies, tailored to *H. vernicosus* as well as its habitats, and will also ensure the effectiveness of protective measures even in the context of future climate change pressures.

Future projections were based on CMIP6 data for two general circulation models—CMCC–ESM2 (Mediterranean-optimized, relevant for Romania) and MIROC6 (broad-range applicability).

## 4. Materials and Methods

### 4.1. Study Area and Site Selection

The study was conducted in Romania, targeting wet peatland habitats classified as Transition mire and quaking bog (NATURA 2000 code: 7140). These ecosystems are thought to be crucial habitats for *H. vernicosus* species because they have oligotrophic to mesotrophic conditions. The site was selected following a comprehensive review of historical records, reports from the literature and internet sources (such as GBIF), as well as recent field observations [28]. This approach included both previously documented localities and potentially suitable but unsurveyed habitats.

GPS coordinates (50 cm or less accuracy) were recorded for each species population (Garmin 64 s). Additionally, aerial photographs of the study site were captured using a DJI Mini 2 drone (SZ DJI Technology Co., Ltd., Shenzhen, China); these images supplemented ground-based observations and made accurate mapping of *H. vernicosus* populations possible by providing high-resolution, landscape-level context for habitat evaluation and spatial analysis.

### 4.2. Field Data Collection

Field surveys were carried out from March to November (2011–2025), which is the optimal period for reliable identification of *H. vernicosus*. In accordance with standard methodologies for rare bryophytes [76,78], permanent plots measuring 5 × 5 m were established in each site and subdivided into units of 1 × 1 m to evaluate the species‘ presence and coverage.

The number of squares varied according to the abundance of the species, and the cover of squares was averaged and extrapolated to the total area extent of the site [78]. “The method of permanent squares” is frequently used and recommended for long-lived species with stable communities on large rocks or on the ground [79].

Species identification was performed in situ using portable magnifiers (×10+) and standard bryological keys–leaf folding and basal leaf color. Due to constraints imposed by legislation, the microscopic confirmation and collection of voucher specimens were not conducted.

For each plot, key ecological parameters were recorded, including habitat type, associated vegetation, hydrological conditions, and some evidence of anthropogenic pressures.

### 4.3. Conservation Status Assessment

The abundance of *H. vernicosus* was estimated by the accepted method of “area covered by the population in 1 m^2^” patches in which the species was present [80]. Although Crum and Anderson (1981) [81] viewed each patch as a distinct ecological individual, it has become challenging to define what an individual is due to the clonal nature of some species [74], as demonstrated by the genetic variations in some populations of *H. vernicosus* [82].

To evaluate population changes and habitat stability, current data were compared with historical observations and records from the same localities enabling the assessment of population dynamics and conservation status over time.

### 4.4. Occurrence Records Processing

We used both original and published distribution records in order to develop spatial distribution models (SDMs) for the varnished hook-moss (*Hamatocaulis vernicosus*), comprising 67 points in total. Occurrences available in the literature were georeferenced using Google Earth Pro v7.3.6.10441, Google Maps (maps.google.com), and military survey maps for Romania with an assumed error of less than 1 km. Before modeling, we rarefied the occurrence dataset using SDMToolbox v2.5 add-on [83] in ArcGIS 10.7.1 and a distance of 1 km to match the spatial resolution of the input predictors, leaving 44 locations for the modeling phase (Figure 2).

### 4.5. Environmental Predictors

We started with packages of 11 variables, describing current and future climate in the area where the species is distributed as well as the elevation (Table 2). Bioclimatic predictors for “current” conditions came from the second-generation baseline (v2.1) [84], and were representative for the 1970–2000 period. The elevation layer used was available from the WorldClim website (www.worldclim.org).

For future conditions, we downloaded corresponding bioclimatic variables based on the Coupled Model Intercomparison Project Phase 6 (CMIP6) [85], where we chose two general circulation models (CMCC–ESM2 and MIROC6). One model was optimized for the Mediterranean region, being developed in Italy by the Euro-Mediterranean Center on Climate Change, and therefore is more relevant for Romania, while the other (MIROC6) was developed in Japan and is representative of a wider range of conditions [86]. We also choose two time periods, one for the middle of the century (2040–2060), centered in 2050, and one for the end of the century (2080–2100), centered in 2090, and two Shared Socioeconomic Pathways (SSPs), SSP245 and SSP585. These would represent two possible trajectories, a more likely outcome (SSP245) and a worst-case scenario (SSP585). All variables were used at a resolution of 30” (~1 km).

### 4.6. Modeling Procedure

We employed ensemble modelling in R (R Core Team, 2022) using the sdm package [87]. SDMs were trained using five methods: GLM (Generalized Linear Models), GAM (Generalized Additive Models), Maxent, BRT (Boosted Regression Trees), and MARS (Multivariate Adaptive Regression Spline), and then projected onto the entire environmental space (as defined above). Before the modeling phase, we first split the environmental rasters into two sets of predictors: (1) one set for training, clipped to the minimum convex polygon enclosing the input features (convex hull) plus a generic buffer of 50 km, and (2) a second set for predictors, clipped to the border of Romania plus a generic buffer of 10 km. This phase as carried out in ArcGIS 10.7.1 [88], using the SDMToolbox v2.5 add-on.

Next we tested multicollinearity among variables using the Variance Inflation Factor (VIF) implemented in the USDM package [89] in R [90] with a VIF threshold of 10 [91], but there were no collinearity problems among variables. We excluded one variable (bio_3) based on low (<1%) contribution to model creation. The final list of predictors used is presented in Table 1.

Settings for modeling included 10,000 random background points, 4-fold cross-validation with 10 repetitions, and a test percentage of 20%. In total 200 models were generated, which were evaluated based on AUC and TSS. The ensembles were generated from models that had AUC values greater than 0.8 or models with TSS values above 0.6.

### 4.7. Post-Modeling Analysis

All ensemble SDMs were reprojected to ETRS89–LAEA in order to extract information on distribution changes in metric units (km^2^). Threshold models were produced for current and future timeframes based on the mean threshold value that maximized the TSS statistic and evaluated using the test data. Distribution changes were evaluated for both global circulation models, the two timeframes, and the two socioeconomic pathways and plotted using the ggplot2 package [92]. To quantify projection uncertainty, we compared results between the two circulation models (ESM2 and MIROC6) for each scenario and period. We calculated suitable area (km^2^) for each ensemble and reported the range (min–max) across circulation models. Model divergence was assessed using Schoener’s D similarity index [75] and calculated from the thresholded maps, where a value of one indicates identical distributions and a value of zero indicates no spatial overlap.

### 4.8. Data Analysis

No generative artificial intelligence (GenAI) has been used in this paper.

## 5. Conclusions

The varnished hook-moss has been found to prefer stable, cool, and moist climatic conditions, as demonstrated by the main climatic factors that influenced its distribution: Annual Mean Temperature, Annual Temperature Range, Precipitation of the Wettest Quarter, and Precipitation of the Coldest Quarter.

Based on climate models, *H. vernicosus* will lose a substantial amount of its distribution space by the end of the century: about 40–50% in the most optimistic scenario and 60% in the most pessimistic one. Furthermore, habitat losses may happen suddenly by the end of 2060 in certain scenarios, with pronounced fragmentation and the emergence of large distribution gaps, especially in the Southern and Western Carpathians.

In light of global climate change, this study strongly supports the development of conservation strategies and significantly advances our knowledge of *H. vernicosus* distribution and vulnerability in Romania. Montane and submontane wetlands must be preserved and protected in order to maintain the microclimate conditions required for the species’ survival.

## Figures and Tables

**Table 1:** Mean performance statistics for each modeling method.

Method	Mean AUC ± SD	Mean TSS ± SD
GLM	0.82 ± 0.41	0.56 ± 0.06
RF	0.76 ± 0.05	0.49 ± 0.06
BRT	0.8 ± 0.05	0.55 ± 0.07
Maxent	0.82 ± 0.04	0.59 ± 0.06
MARS	0.79 ± 0.08	0.55 ± 0.1

**Table 2:** List of environmental variables used for modeling.

Code	Name	Relative Importance Based on AUC (%)
bio_1	Annual Mean Temperature	56.4
bio_2	Mean Diurnal Range (mean of monthly (max temp–min temp))	17.6
bio_3	Isothermality (BIO2/BIO7) (×100) ^1^	-
bio_4	Temperature Seasonality (standard deviation × 100)	8.2
bio_7	Temperature Annual Range (BIO5-BIO6)	16
bio_10	Mean Temperature of Warmest Quarter	12.1
bio_12	Annual Precipitation	12.4
bio_16	Precipitation of Wettest Quarter	10.3
bio_18	Precipitation of Warmest Quarter	10.6
bio_19	Precipitation of Coldest Quarter	6.8
elev	Digital Elevation Model (DEM)	11.4

^1^ excluded from the modeling phase based on low contribution.

## Data Availability

The original contributions presented in this study are included in the article/Supplementary Material. Further inquiries can be directed to the corresponding authors.

## Supplementary Materials

The following supporting information can be downloaded at https://www.mdpi.com/article/10.3390/plants14213354/s1, Figure S1: Predicted distribution range changes for the varnished hook-moss. Tabel S1: Range of projected suitable areas (km^2^) and spatial overlap (Schoener’s D) between global circulation models for each scenario and time period.

## Abbreviations

The following abbreviations are used in this manuscript:

BP	Hungarian National Museum Public Collection Centre, Budapest, Hungarian Natural History Museum, Hungary; Botanical Department, Herbarium BP, Bryophyte herbarium, *S*–*Sphagnum* collection
BRNU	Masaryk University, Faculty of Science, Department of Botany and Zoology, Brno, Czech Republic; Herbarium BRNU
