# Supplementary material for: Climate Change Projected Effects on Hamatocaulis vernicosus Occurrence in Romania
Source: Plants (Basel). 2025 Oct 31;14(21):3354. doi: 10.3390/plants14213354 (PMC12609563; doi:10.3390/plants14213354)
Supplement: Supplementary file 1 [file plants-14-03354-s001.zip › Figure S1. plants-3861144-supplementary.pdf]

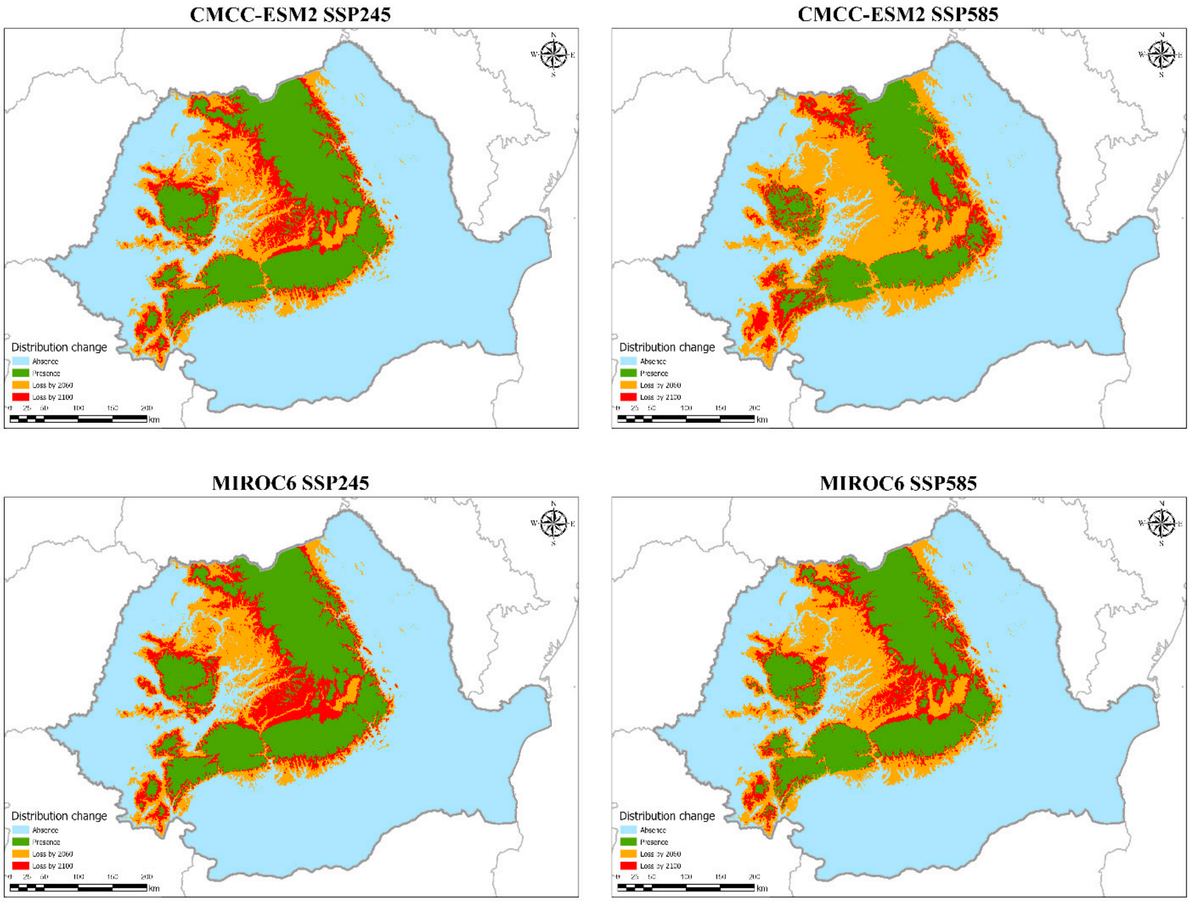

Figure S1 Predicted distribution range changes for the varnished hook-moss (*Hamatocaulis vernicosus*) by 2100 based on the two global circulation models used
